# Ambulatory Pediatric Surveillance of Hand, Foot and Mouth Disease as Signal of an Outbreak of Coxsackievirus A6 Infections, France, 2014–2015

**DOI:** 10.3201/eid2211.160590

**Published:** 2016-11

**Authors:** Audrey Mirand, François Vié le Sage, Bruno Pereira, Robert Cohen, Corinne Levy, Christine Archimbaud, Hélène Peigue-Lafeuille, Jean-Luc Bailly, Cécile Henquell

**Affiliations:** Centre Hospitalier Universitaire de Clermont-Ferrand, Clermont-Ferrand, France (A. Mirand, B. Pereira, C. Archimbaud, H. Lafeuille, J.-L. Bailly, C. Henquell);; Université d’Auvergne, Clermont-Ferrand (A. Mirand, C. Archimbaud, H. Peigue-Lafeuille, J.-L. Bailly, C. Henquell);; Association Française de Pédiatrie Ambulatoire, Bagnols-sur-Cèze, France (F. Vié le Sage);; Association Clinique et Thérapeutique Infantile du Val de Marne, Saint Maur des Fossés, France (R. Cohen, C. Levy);; Université Paris Est, Créteil, France (R. Cohen, C. Levy)

**Keywords:** enterovirus A, human coxsackievirus infections, sentinel surveillance, hand, foot and mouth disease, ambulatory, pediatric, surveillance viruses, France

## Abstract

The clinical impact of enteroviruses associated with hand, foot and mouth disease (HFMD) is unknown outside Asia, and the prevalence of enterovirus A71 (EV-A71) in particular might be underestimated. To investigate the prevalence of enterovirus serotypes and the clinical presentations associated with HFMD in France, we conducted prospective ambulatory clinic–based surveillance of children during April 2014–March 2015. Throat or buccal swabs were collected from children with HFMD and tested for the enterovirus genome. Physical examinations were recorded on a standardized form. An enterovirus infection was detected in 523 (79.3%) of 659 children tested. Two epidemic waves occurred, dominated by coxsackievirus (CV) A6, which was detected in 53.9% of enterovirus-infected children. CV-A6 was more frequently related to atypical HFMD manifestations (eruptions extended to limbs and face). Early awareness and documentation of HFMD outbreaks can be achieved by syndromic surveillance of HFMD by ambulatory pediatricians and rapid enterovirus testing and genotyping.

## Abstract 2

Outbreaks can be detected by syndromic surveillance, rapid enterovirus testing, and genotyping.

Although mostly asymptomatic or self-limited, enterovirus infections comprise a wide spectrum of clinical manifestations in children, which can require medical attention. Periodically, the emergence of an enterovirus serotype is associated with outbreaks of more serious disease resulting in serious illness and even death. Recent examples are the emergence of enterovirus A71 (EV-A71), which was responsible for large hand, foot and mouth disease (HFMD) outbreaks associated with rare but severe rhombencephalitis in Asia, and an EV-D68 epidemic associated with severe respiratory infections (*1*,*2*). Monitoring enterovirus infections and providing laboratory confirmation of the serotypes associated with different clinical presentations are of value for the early detection and awareness of emerging enterovirus infections (*3*,*4*).

EV-A71 is considered to be the most important neurotropic enterovirus in Southeast Asia countries, and EV-A71 vaccines have been developed in China (*5*,*6*). EV-A71 infections, along with other enterovirus serotypes belonging to the species *Enterovirus A* (EV-A) (*7*), are mainly associated with HFMD, which is characterized in children by a brief febrile illness and typical rash, with or without mouth ulcers (*8*). EV-A71 and coxsackievirus (CV) A16 were the most frequent serotypes involved in HFMD outbreaks throughout Asia during 1998–2010 (*1*,*9*). In the past 5 years, however, CV-A6 has emerged as a new important pathogen worldwide (*10*–*19*), and several studies have documented the more severe and extensive dermatologic presentations of CV-A6 HFMD (*16*,*20*–*25*). Surveillance of HFMD could lead to better detection of the upsurge of EV-A71 or another serotype associated with severe or distinct clinical features. In Western countries, surveillance of enterovirus infections is undertaken by virology laboratories and is thus restricted to enterovirus-infected persons admitted to hospitals (*19*,*26*). Children with HFMD or herpangina are usually evaluated and managed in ambulatory settings, and virologic investigations are rarely performed. Consequently, a clear gap exists in the knowledge of the epidemiology and clinical impact of HFMD and herpangina and of the enteroviruses involved in countries outside Asia.

We set up a local surveillance system run by pediatricians in ambulatory care settings that was effective in detecting HFMD outbreaks and the associated enterovirus serotypes (*13*). We have now extended this surveillance to cover the whole of France. The objectives of this study were to describe the epidemiology of enterovirus serotypes associated with HFMD and herpangina in France and to compare the clinical characteristics of HFMD and herpangina according to enterovirus serotypes.

## Methods

### Study Population and Design 

The study was a 1-year prospective investigation of children with HFMD or herpangina who were seen by their pediatrician during April 2014–March 2015. The sentinel surveillance was performed by 47 pediatricians selected from among 118 volunteers by stratified sampling in different regions of France; 20 of the 22 French administrative regions were represented (Technical Appendix). Sentinel pediatricians were requested to collect throat or buccal swab specimens from children with clinically diagnosed HFMD/herpangina. HFMD was defined by the presence of >2 of the following signs: buccal or peribuccal ulcers; eruption on palms, soles, buttocks, knees, or elbows; or a generalized eruption. Herpangina was defined by the presence of oral ulcers predominating on the posterior part of the buccal cavity. A standardized case report form collected anonymized information on the patient’s demographics (e.g., birth date and sex); clinical signs at presentation, including fever, eruption type, and localization (e.g., palms, soles, buttocks, knees, elbows, lower limbs, upper limbs, generalized, or any other localization), buccal or peribuccal ulcers, gingivostomatitis, herpangina, and digestive/respiratory/ear, nose, and throat/neurologic signs; the onset date of the disease; and the date of sample collection. Environmental data (e.g., number of siblings, attendance at school or a daycare center, and ill contacts) were also recorded. Fever was defined as a rectal temperature >38°C. On the basis of the items checked, typical HFMD was defined as the presence of >2 of the following signs, as listed in the HFMD definition of the World Health Organization (*8*): oral ulcerations, eruption on palms, soles, buttocks, knees, or elbows. Clinical signs were considered to be atypical if eruption occurred at anatomic sites not listed in the World Health Organization HFMD definition or if it was generalized.

Written informed consent was obtained from the parents or guardians of all participants, none of whom received a stipend. The study was approved by the Ethics Review Committee of the University Hospital of Clermont-Ferrand, France (reference AU1098), and by the French National Agency for the Safety of Medicines and Health Products (reference 140021-B41).

### Sample Collection

Throat or buccal specimens were collected with a flocked swab placed in a universal virus transport system (Copan Italia, s.p.a., Brescia, Italy). After the sampling, swabs were conserved at 2° –8°C and sent weekly to the National Reference Laboratory for Enterovirus and Parechovirus (Clermont-Ferrand, France) for enterovirus testing.

### Diagnosis of Enterovirus Infection and Molecular Typing of Strains in Clinical Specimens

**Figure 1:**
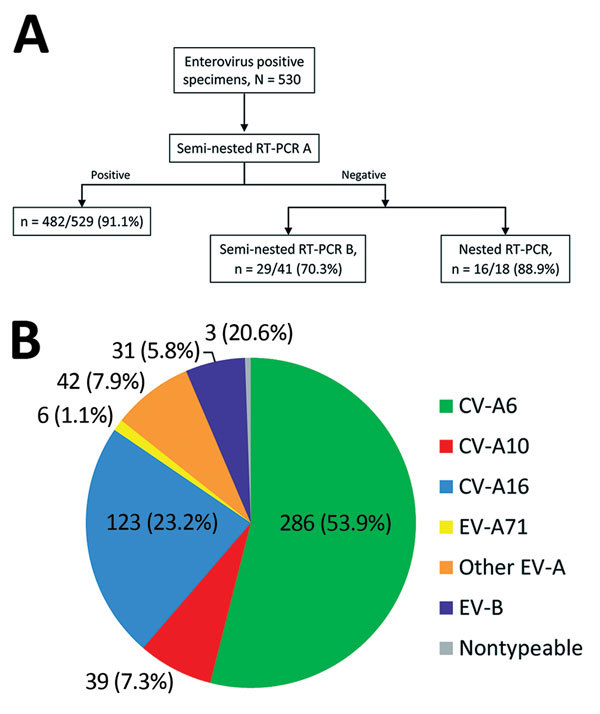
Methodologic approach for enterovirus genotyping and distribution of types associated with hand, foot and mouth disease and herpangina, France, April 2014–March 2015. A) Semi-nested reverse transcription PCR (RT-PCR) A using primers specifically developed for enterovirus types belonging to the EV-A species was first performed for all clinical samples except 1. For this sample, the viral load was low, and the nested RT-PCR described by Nix et al. (*27*) was performed directly. If the semi-nested RT-PCR A was negative, the genotyping was alternatively performed by a semi-nested RT-PCR B with primers specific to the EV-B species (*28*) or a nested RT-PCR (*27*). B) Among other EV-A species, 5 different types were identified: coxsackievirus (CV) A4, n = 18; CV-A8, n = 16; CV-A2 and CV-A5, n = 5 each; and CV-A12, n = 1. Among EV-B species, 12 different types were identified: echovirus (E) 16 (E-16) and E-18 (n = 5 each); E-11 and coxsackievirus B3 (CV-B3; n = 4 each); CV-B1, CV-B2, CV-B4, CV-A9, and E-6 (n = 2 each); and E-3, E-5, and E-25 (n = 1 each). EV-A, *Enterovirus A*; EV-A71, enterovirus A71; EV-B, *Enterovirus B*; RT-PCR, reverse transcription PCR.

Viral RNA was extracted from 200 µL of the universal virus transport medium on the NucliSens easyMAG automated system (bioMérieux, Marcy l’Etoile, France) by the specific B protocol (elution volume 50 µL). Enterovirus diagnosis was performed by real-time reverse transcription PCR (RT-PCR) (Enterovirus R-gene, bioMérieux). Molecular typing (Figure 1, panel A) was first performed by a semi-nested RT-PCR with primers specifically developed for EV-A types (RT-PCR A) that targets the viral protein (VP) 3–VP1 coding region of the enterovirus genome. The first round of the RT-PCR EV-A assays were performed in a final volume of 25 µL with primers HEVAS1405 (5′-GGNTCNTTYATGGCNACNGGNAARATG-3′, location 1,405–1,531 relative to the genome of CV-A6 Gdula strain) and EVAR2C (5′-CGGTGYTTGCTCTTGAACTGCATG-3′, location 4,439–4,416) at a final concentration of 0.5 µmol/L, each by using the One-Step RT-PCR kit (QIAGEN, Courtaboeuf, France). The amplification program was as follows: 1 cycle of 30 min at 50°C; 1 cycle of 15 min at 95°C; 41 cycles of 30 s at 94°C, 50 s at 55°C, and 2 min at 72°C; and a final cycle of 10 min at 72°C. The second round RT-PCR A assays were performed in a final volume of 50 µL by using the Taq polymerase Kit (QIAGEN) and contained 5 µL of the first RT-PCR A amplicons and primers HEVAS1405 and HEVAR2429 (5′-GTNGGRTANCCRTCRTARAACC-3′, location 2,450–2,429) at a final concentration of 0.4 µmol/L. The reaction was run under the following conditions: 1 cycle of 2 min at 94°C; 39 cycles of 15 s at 94°C, 50 s at 54°C, and 50 s at 72°C; and a final cycle of 5 min at 72°C. If results were negative, a semi-nested RT-PCR was performed with primers specific for the species *Enterovirus B* (EV-B), HEVBS1695/EV2C (first round) and HEVS1695/HEVBR132 (second round) (*27*). The amplification programs were the same as those described except for the hybridization step, which was performed at 58°C. Alternatively, genotyping was attempted with nonspecies-specific primers to amplify the partial VP1 gene (*28*). Visible RT-PCR products after gel electrophoresis were purified and subjected to nucleotide sequencing with the same primers used for the amplification for the semi-nested RT-PCR A or, as previously described (*27*,*28*), by using the BigDye Terminator v1.1 Cycle Sequencing Kit (Thermo Fisher Scientific, Ville-bon sur Yvette, France). The sequencing was performed on an ABI3500Dx genetic analyzer (Thermo Fisher Scientific). Virus identification was performed by BLAST analysis (http://www.ncbi.nlm.gov/BLAST) and confirmed by phylogenetic analysis (*13*). The results were prospectively sent to the attending pediatricians, and monthly feedback on the overall findings of the surveillance was provided.

### Phylogenetic Analyses

To investigate the spatiotemporal relationships among virus variants, we compared the nucleotide VP1 sequences assigned to the enterovirus serotype CV-A6 with homologous sequences available in public databases. We discarded redundant sequences from the final alignment of 238 sequences of 369 nt (i.e., 159 sequences determined in this study and 79 publicly available sequences). The phylogenetic relationships were inferred using a Bayesian method implemented in the BEAST package version 1.8 (http://beast.bio.ed.ac.uk). The uncorrelated lognormal molecular clock was employed with a flexible Bayesian skyline plot coalescent prior and the general time reversible model of nucleotide substitution. The Markov chain Monte Carlo were run for 200 million generations. We calculated maximum clade credibility trees by using the Tree Annotator program version 1.5.4 in BEAST. Topological support was assessed by calculating the posterior probability (pp) density for each node. All sequences were deposited into the GenBank database (accession nos. LT595894–LT596052). To characterize the EV-A71 strains, we compared partial VP1 sequences with reference sequences for genogroups A–F and subgenogroups B0–B5 and C1–C5. Phylogenetic analysis was performed with the neighbor-joining method and the Tamura-Nei model of sequence evolution implemented in MEGA6 software (http://www.megasoftware.net).

### Statistical Analyses

We performed statistical analyses with Stata 13 software (StataCorp LP, College Station, TX, USA). The tests were 2-sided, with a type I error set at α = 0.05. Patient characteristics were presented as mean (±SD) for continuous data (assumption of normality assessed by the Shapiro–Wilk test) and as the number of patients and associated percentages for categorical parameters. We classified patients according to statistical distribution and epidemiologic relevance into 4 age groups: 1) <1 year old, 2) >1 year old, 3) 2 to <3 years old, and 4) >3 years old. We compared the independent groups (i.e., age groups and CV-A6 infections [yes/no]) by χ^2^ or Fisher exact test for categorical variables and by analysis of variance (ANOVA) or Kruskall-Wallis test for quantitative parameters (assumption of homoscedasticity analyzed by Fisher-Snedecor test). When appropriate (ANOVA or Kruskall-Wallis; p<0.05), we performed post hoc tests (Tukey-Kramer after ANOVA and Dunn after Kruskall-Wallis test) for multiple comparisons, particularly for comparisons between classes of age. Multivariate analyses (logistic regression for dichotomous independent variable) were performed to take into account adjustment on covariates fixed according to univariate results and clinical relevance (i.e., age at enrolment and time between onset and consultation).

## Results

Of the 659 children enrolled in the study, 523 (79.3%) had an enterovirus infection. Ten patients experienced 2 episodes of HFMD/herpangina during the study period and had a specimen collected; 2 successive enterovirus infections associated with different serotypes occurred in 7 children at intervals of 3 weeks to 5.5 months. For the other 3 patients, only 1 episode was associated with an enterovirus infection. 

**Figure 2:**
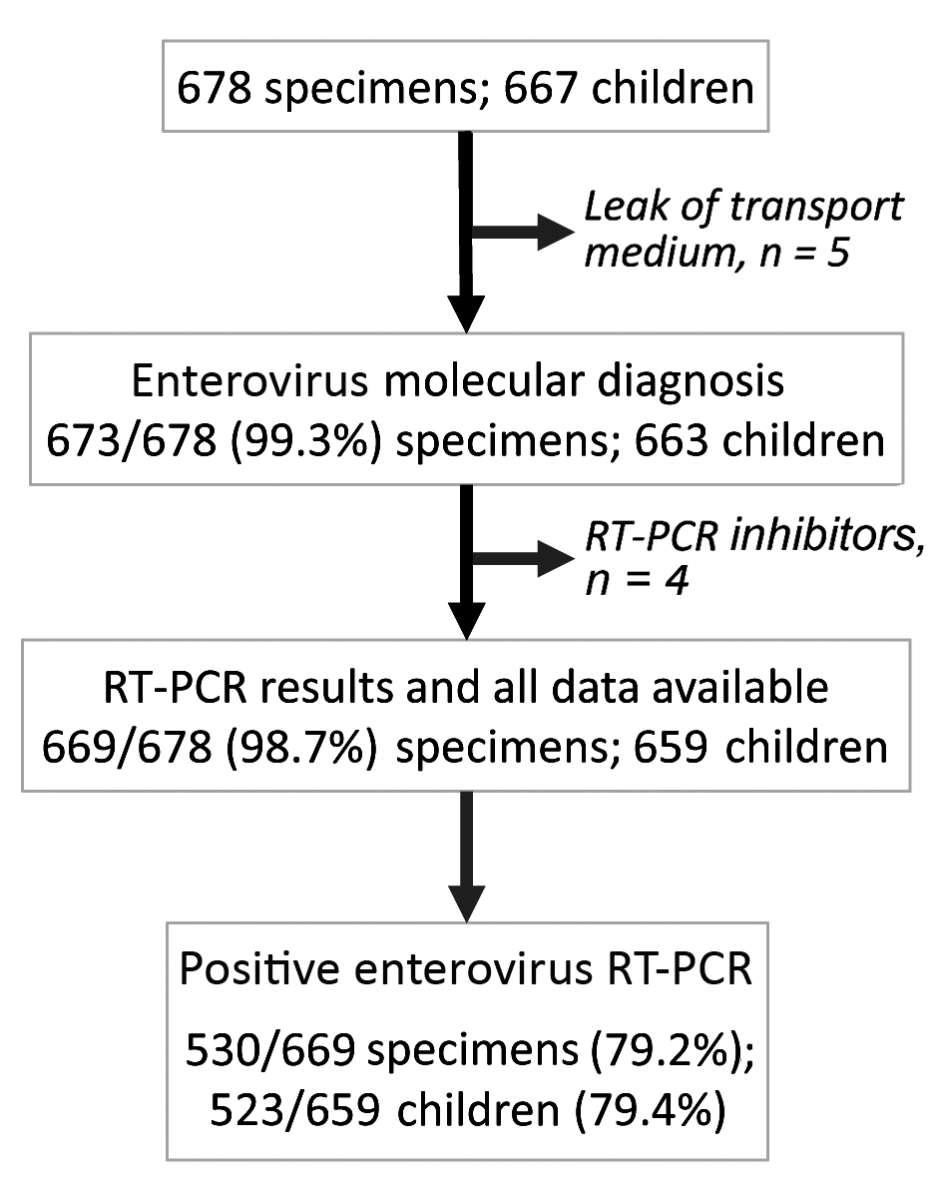
Participant flow diagram of enterovirus testing for the surveillance of hand, foot and mouth disease and herpangina, France, April 2014–March 2015. RT-PCR, reverse transcription PCR.

**Figure 3:**
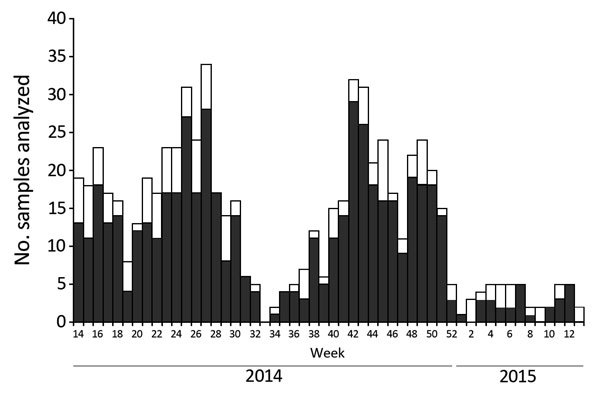
Weekly distribution of enterovirus infections associated with hand, foot and mouth disease and herpangina, France, April 2014–March 2015. Bar sections represent the number of enterovirus-positive (dark gray) and -negative (white) samples analyzed.

Overall, 669 specimens were analyzed, of which 530 (79.2%, 95% CI 75.9–82.2) tested positive for enterovirus (Figure 2). Mean patient enterovirus positivity rate for participating pediatricians was 75.1% (range 50.0%–88.9%). Enterovirus-associated HFMD/herpangina showed biannual peaks of activity in early summer (weeks 25–27) and in autumn (week 42) (Figure 3). 

An enterovirus serotype was identified for 527/530 (99.4%) of proven infections. The most frequent EV-A serotype was CV-A6 (286/530, 53.9%) followed by CV-A16 (123/530, 23.2%), CV-A10 (39/530, 7.3%), CV-A4 (18, 3.3%), CV-A8 (16, 3.0%), CV-A2 (5, 0.9%), and CV-A5 (5, 0.9%); 1 infection was CV-A12 (Figure 1, panel B). Twelve EV-B serotypes were identified: echovirus 16 (E-16) and E-18 (5 each); E-11 and coxsackievirus B3 (CV-B3) (4 each); CV-B1, CV-B2, CV-B4, CV-A9, and E-6 (2 each); and E-3, E-5, and E-25 (1 each). CV-A6 was predominant during both epidemic waves. Six EV-A71 infections were detected, most associated with typical HFMD (5/6, 83.3%). One patient had generalized eruption. Fever was reported for only 2/6 patients. EV-A71 strains belonged to subgenotypes C2 (n = 5) and C4 (n = 1). The C4 strain was identified in a 3-year-old child from Guangzhou, China, who was on a visit to France (data not shown).

The mean age of enterovirus-infected children was 2.1 years (range 1 month–10.5 years). The highest rate of infections was observed in children 1–2 years of age. Fever was reported in (397/530, 74.9%) of enterovirus-infected children. Cutaneous eruption was observed in 456/530 (86%) children and affected, in decreasing order, the palms, soles, buttocks, and elbows. HFMD was the predominant clinical presentation (342/530, 64.5%). Herpangina was reported in 304/530 (57.4%) of cases and was associated with clinical signs of HFMD in 241/304 (79.2%). Lesions were also frequently observed on the limbs (188/530, 35.4%) and the face (perioral and earlobes, 161/530, 30.4%). Atypical HFMD was observed in 247/530 (46.6%) children (Table 1). The proportions of enterovirus-infected children were not significantly different between children presenting with typical HFMD (95/116, 81.9%) or atypical HFMD (247/281, 87.9%). 

**Table 1:** Demographic and clinical features of patients with CV-A6 infections compared with those with non–CV-A6 infections, France, April 2014–March 2015*

Characteristic	All enterovirus infections, n = 530	CV-A6 infections, n = 286	Non–CV-A6 infections, n = 210	p value†
Age at enrollment, y, mean (SD)	2.1 (1.41)	1.69 (0.93)	2.53 (1.78)	**<0.001**
Male sex, no. (%)	290/523 (55.4)	152/281 (54.1)	123/209 (58.9)	0.29
Time between onset and consultation, d, mean (SD)‡	1.92 (1.35)	2.13 (1.49)	1.63 (1.06)	**<0.001**
Signs and symptoms, no. (%)				
Fever	397 (74.9)	220 (76.9)	147 (70.0)	0.08
Oral ulcerations	224 (42.3)	101 (35.3)	118 (56.2)	**<0.001**§
Gingivostomatitis	79 (14.9)	36 (12.6)	42 (20)	**0.02**
Eruption	456 (86.0)	268 (93.7)	163 (77.6)	**<0.001**§
Vesicular eruption	355 (70.3)	226/278 (81.3)	119/197 (60.4)	**<0.001**§
Nonvesicular eruption	160 (30.2)	94 (32.9)	53 (25.2)	0.07
Localizations of eruption, no. (%)				
Palms	308 (58.1)	190 (66.4)	114 (54.3)	**0.006**§
Soles	279 (52.6)	160 (55.9)	111 (52.9	0.49
Buttocks	251 (47.4)	171 (59.8)	73 (34.8)	**<0.001**§
Elbows / knees	133 (25.1)	90 (31.5)	40 (19.0)	**0.002**§
Lower limbs	170 (32.1)	131 (45.8)	27 (12.9)	**<0.001**§
Upper limbs	119 (22.5)	83 (29.0)	24 (11.4)	**<0.001**§
Generalized eruption	46 (8.7)	27 (9.4)	14 (6.7)	0.27
Trunk	23 (4.3)	13 (4.5)	5 (2.4)	0.20
Face, including perioral ulcerations	161 (30.4)	134 (46.9)	24 (11.4)	**<0.001**§
Diagnosis, no. (%)				
Typical HFMD¶	95 (17.9)	32 (11.2)	62 (29.5)	**<0.001**§
Atypical HFMD	247 (46.6)	181 (63.3)	59 (28.1)	**<0.001**§
Herpangina	304 (57.4)	165 (57.7)	110 (52.4)	0.24
Herpangina alone	63 (11.9)	15 (5.2)	40 (19.0)	**<0.001**§
Other signs, no. (%)				
Digestive signs	61 (11.5)	38 (13.3)	15 (7.1)	**0.03**§
Ear, nose, and throat signs	54 (10.2)	27 (9.4)	26 (12.4)	0.29
Respiratory signs	15 (2.8)	6 (2.1)	7 (3.3)	0.39


*Non–CV-A6 infections only include infections by another type belonging to the enterovirus A species. CV-A6, coxsackievirus A6; HFMD, hand, foot and mouth disease. †Statistical analyses were performed to compare clinical characteristics of CV-A6 and non–CV-A6 infections. Significant p values (p<0.05) are indicated in bold type. ‡Time from symptom onset and consultation was available for 525 episodes of enterovirus infection. §Indicates significant differences between CV-A6 infections and non–CV-A6 infections by multivariate analyses. ¶Typical HFMD was defined as the presence of >2 of the following signs: oral ulcerations, eruption on palms, soles, buttocks, knees, or elbows, excluding any other localization. Atypical HFMD was defined by the presence of >2 of those signs plus the involvement of another anatomic site.

“Eczema coxsackium or herpeticum” was reported in 8 children, of whom 7 had a CV-A6 infection and 1 a CV-A10 infection. An eruption mimicking a Gianotti-Crosti syndrome caused by different enterovirus serotypes (CV-A6 [n = 3]; CV-A10 [n = 1]; or CV-A16 [n = 1]) was reported in 5 of these 8 children. Exposure to ill contacts was reported for 155/469 (33%) enterovirus-infected children with available environmental data, a proportion significantly higher than that among non–enterovirus-infected children (25/127, 19.7%; p = 0.004).

Patients with CV-A6 HFMD/herpangina were significantly younger than patients with other EV-A serotypes (p<0.001) (Table 1). The clinical features of CV-A6-associated HFMD were also significantly different. Atypical HFMD was more frequently reported in CV-A6-infected children (181/286, 63.3%) than in children infected with other enterovirus serotypes: CV-A16 (42/123, 34.1%), CV-A10 (14/39, 35.8%), CV-A4 and E-16 (2 cases each), and CV-A12, E-6, E-11, E18, or CV-B4 (1 case each). Typical HFMD or herpangina alone were significantly more frequent in children infected by the other EV-A serotypes (p<0.001) (Table 1). The most frequent serotypes associated with typical HFMD were CV-A16 (54/95, 56.8%) and CV-A6 (32/95, 33.7%), followed by EV-A71 (n = 4), CV-A8 and CV-A10 (n = 2 each), and E-5 (n = 1). The most frequent serotypes associated with herpangina alone were CV-A6 (14/63, 22.2%), CV-A4 (11/63, 17.4%), CV-A8 (10/63, 15.9%), and CV-A10 (8/63, 12.7%). The differences between CV-A6 infections and non–CV-A6 infections remained statistically significant by multivariate analyses. 

Atypical HFMD was less frequent in patients <1 year of age than in children 1–2 and >3 years of age. Herpangina alone was more frequent in children <1 year of age than in older children (Table 2).

**Table 2:** Demographic and clinical features associated with CV-6 infections in 4 age groups of patients, France, April 2014–March 2015*

Characteristic	<1 y, n = 63	1–2 y, n = 146	2–3 y, n = 50	>3 y, n = 26	p value†
Male sex, no. (%)	35/62 (56.4)	80/146 (55.5)	26/49 (53.1)	10/25 (40	0.52
Time between onset and consultation, d, mean (SD)‡	1.84 (1.31)	2.27 (1.43)	1.87 (1.48)	2.46 (2.02)	0.09
Signs and symptoms, no. (%)					
Fever	54 (85.7)	105 (71.92)	38 (76)	22 (84.6)	0.13
Oral ulcerations	17 (27)	53 (36.3)	19 (38)	12 (46.1)	0.32
Perioral ulcerations	18 (28.57)	67 (45.9)	25 (50)	15 (57.7)	**0.003**
Eruption	53 (84.1)	143 (97.9)	45 (90)	26 (100)	**0.001**
Vesicular eruption	43 (71.7)	120 (83.9)	41 (83.7)	21 (84)	0.21
Nonvesicular eruption	20 (31.7)	55(37.7)	11 (22)	7 (26.9)	0.20
Localizations of eruption, no. (%)					
Palms	41 (65.1)	96 (65.75)	33 (66)	20 (76.9)	0.71
Soles	34 (54)	86 (58.9)	24 (48)	16 (61.5)	0.53
Buttocks	29 (46)	100 (68.5)	30 (60)	12 (46.1)	**0.009**
Elbows or knees	15 (23.8)	49 (33.6)	16 (32)	10 (38.5)	0.46
Lower limbs	20 (31.8)	75 (51.4)	26 (52)	10 (38.5)	**0.04**
Upper limbs	8 (12.7)	55 (37.7)	17 (34)	3 (11.5)	**<0.001**
Generalized eruption	9 (14.3)	14 (9.6)	3 (6)	1 (3.9)	0.41
Trunk	ND	ND	ND	ND	ND
Face, including perioral ulcerations	21 (33.3)	70 (48)	27 (54)	15 (57.7)	0.07
Diagnosis, no. (%)					
Typical HFMD§	10 (15.9)	17 (11.6)	3 (6)	2 (7.7)	0.38
Atypical HFMD	30 (47.6)¶	100 (68.5)¶	32 (64)	19 (73.1)¶	**0.02**
Herpangina	45 (71.4)	76 (52.1)	29 (58)	15 (57.7)	0.08
Herpangina alone	9 (14.3)	2 (1.4)	4 (8)	0	**0.001**
Other signs, no. (%)					
Digestive signs	7 (11.1)	16 (11)	10 (20)	5 (19.2)	0.28
Ear, nose, and throat signs	4 (6.4)	18 (12.3)	3 (6)	2 (7.7)	0.41
Respiratory signs	ND	ND	ND	ND	ND


*For 1 patient, age was not known. CV-A6, coxsackievirus A6; HFMD, hand, foot and mouth disease; ND, not determined because of low sample size (<15 patients total). †Significant p values (p<0.05) are indicated in bold type.  ‡Time from symptom onset and consultation was available for 525 episodes of enterovirus infection. §Typical HFMD was defined as the presence of >2 of the following signs: oral ulcerations, eruption on palms, soles, buttocks, knees, or elbows, excluding any other localization. Atypical HFMD was defined by the presence of >2 of those signs plus the involvement of another anatomic site. ¶Indicates significant differences between age groups.

**Figure 4:**
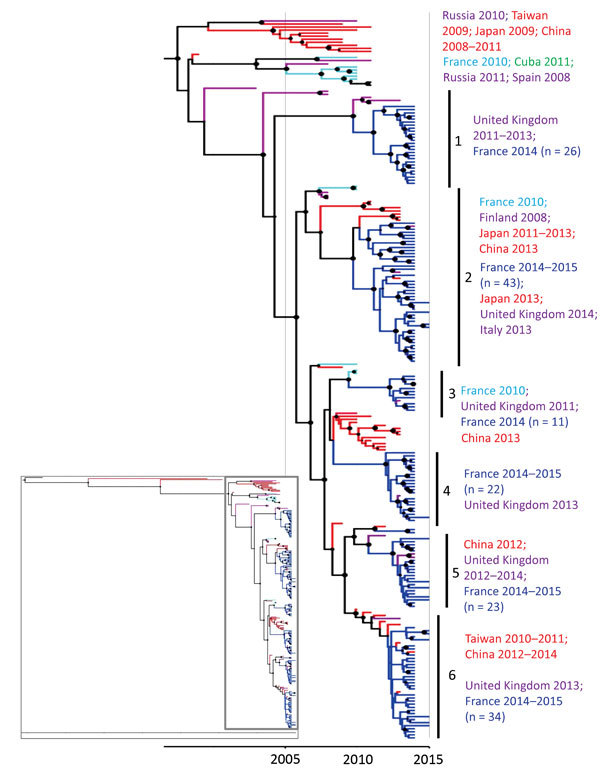
Phylogenetic tree based on partial viral protein (VP1) coding sequences of coxsackievirus (CV) A6, France, April 2014–March 2015. The maximum credibility tree is inferred with the partial VP1 sequence (369 nt, position 2,441–2,808 relative to the Gdula CV-A6 prototype strain). The phylogenetic relationships were inferred with a Bayesian method by using a relaxed molecular clock model. The tree was reconstructed using Figtree version 1.4.2 (http://tree.bio.ed.ac.uk/software/figtree). For clarity, the sequence names are not included in the tree. Circle sizes are proportional to posterior probability. Each tip branch represents a sampled virus sequence. The continents/countries where the virus strains were sampled are indicated by different colors: Europe, purple; France 2010, light blue; France 2014–2015, dark blue; the Americas, green; Asia, red. The inset shows the complete tree, with the box indicating the portion enlarged for clarity.

The CV-A6 strains sampled in France in 2014 and 2015 were grouped in 6 co-circulating lineages supported by high posterior probability values (Figure 4). The nucleotide identities within lineages ranged from 95.3% to 98.9% (98.3%–99.5% amino acid identities). Between lineages, the nucleotide identities ranged from 91.9% to 96.3% (97.5%–98.4% amino acid identities), the highest divergence being observed between lineages 1 and 2 and the lowest between 5 and 6. In lineage 1, the virus strains collected in France were temporally distantly related to viruses collected 1–3 years earlier in the United Kingdom. In the other 5 lineages, the CV-A6 virus strains sampled in France displayed close temporal relationships to viruses recovered in other countries in Europe since 2010. In the lineages 2 and 6, close genetic and temporal relationships were also estimated between virus strains recovered in France and countries in Asia. The 2014 CV-A6 viruses in lineage 3 were genetically related to those recovered in 2010 in France.

## Discussion

This prospective ambulatory clinic–based surveillance of HFMD/herpangina in children revealed the global effect of these diseases in France. Data were collected from a standardized report of clinical signs, which provided a comprehensive description of the clinical characteristics of these syndromes associated with different enterovirus serotypes. During April 2014–March 2015, CV-A6 infections were associated with HFMD in 74.4% cases and herpangina in 57.7% of cases. These proportions were inverted during the 2010 HFMD outbreak in central France: 50% for HFMD cases and 70% for herpangina (*13*). In addition, the dermatologic presentation of CV-A6 HFMD cases was more frequently unusual (63.3%), with eruptions extending beyond the typical sites of HFMD (i.e., soles, palms, buttocks, and knees or elbows) than in HFMD cases caused by other enterovirus serotypes. Our findings suggest that the clinical presentation of CV-A6 infections in France shifted to atypical HFMD during 2010–2014, as observed in China during 2008–2013 (*18*). In our study, the co-circulation of 6 virus lineages in 2014 is consistent with the hypothesis of multiple introductions of genetically distinct CV-A6 strains. In addition, a genetic analysis of complete CV-A6 genomes showed that strains collected during the 2012–2013 outbreak in Shanghai, China, were recombinant compared with strains collected before 2009 and were more frequently associated with generalized rash (*29*).

Comparative analyses of whole virus genomes should be expanded on large sequence data derived from prospective epidemiologic studies to investigate whether the changes in the clinical features of CV-A6 infections reported here are determined by viral factors. The relationship between CV-A6 and atypical HFMD has been reported in earlier studies that described frequent unusual morphology or extent of cutaneous findings (*15*,*20*–*25*,*30*), such as “eczema coxsackium,” Gianotti-Crosti-like eruption, and purpuric eruption (*21*). However, these studies were either retrospective, focused on severe or atypical HFMD, or performed in dermatology pediatric centers, which might have biased the clinical spectrum of CV-A6 HFMD cases toward more severe or atypical presentations. Although we cannot exclude the possibility that pediatricians were more prone to include children with unusual presentations of HFMD, our study confirms that CV-A6 is more frequently associated with atypical HFMD even in an ambulatory setting. 

Of note, atypical HFMD was reported in 66/244 (27%) of non–CV-A6-associated HFMD cases. This result might be attributable to the definition of atypical HFMD we used, which was, in contrast to that of typical HFMD (*8*), the involvement of a nontypical anatomic site for HFMD. The lack of a consensus definition of atypical HFMD and the fact that the collected clinical data vary between studies hamper rigorous comparisons between them. Further investigations based on prospective ambulatory clinic–based surveillance of HFMD are needed to determine whether our observation is attributable to a specific increase in the circulation of CV-A6 or to a global change in the transmission of enterovirus strains. The documentation of unusual presentations of HFMD by enterovirus genotyping is useful for detecting the emergence of a new serotype with distinct clinical features.

The clinical courses of typical and atypical HFMD seemed similar in our cohort because no complications were reported. The extensive or unusual nature of the cutaneous manifestations of CV-A6 HFMD does not seem to increase the risk for severe systemic illness (*21*). Although to a lesser extent than with EV-A71, severe CV-A6 HFMD cases, defined by the presence of neurologic signs (e.g., meningitis, encephalitis, acute flaccid paralysis, and seizures) or cardiopulmonary signs, have been reported, with a frequency ranging from 3.6 % to 18.2% during the recent CV-A6 outbreaks in China (*18*,*31*–*34*). Meningitis rather than encephalitis was more frequently associated with severe CV-A6 HFMD (*32*,*33*). However, clinicians should be aware of the potential neurotropism of all EVs. As exemplified by EV-A71 outbreaks (*35*) and more recently EV-D68 outbreaks (*2*), the high rate of circulation of CV-A6, either symptomatic or asymptomatic, can lead to the more frequent observation of this serotype in association with neurologic signs.

As is the case with many common and self-limited illnesses, the children in our study might not have attended all medical consultations, thus rendering the surveillance incomplete. The comprehensive recruitment of all children with HFMD/herpangina is time-consuming and not feasible in the routine practice of ambulatory pediatrics, which might have resulted in a lower inclusion rate. We do not have recorded long-term follow-up or the specific CV-A6–associated clinical entities described by Mathes et al. (*13*), and we were not able to assess the occurrence of onychomadesis or desquamation of the extremities, which have been frequently associated with CV-A6 outbreaks (*9*–*11*,*15*,*20*,*32*).

This study contributes to a more comprehensive view of the epidemiology of HFMD/herpangina in France and the clinical spectrum of HFMD/herpangina associated with enterovirus, in particular with CV-A6. The often unusual presentation of HFMD can be challenging for clinicians, and this study might therefore help improve the differential diagnosis of HFMD by primary care physicians and the detection of future HFMD outbreaks. Syndromic surveillance of HFMD/herpangina by pediatricians in ambulatory setting with prospective and standardized collection of clinical data in combination with enterovirus testing and genotyping are useful for monitoring the epidemiology of enterovirus infections, for the timely detection of peaks of highest activity, and for determining the enterovirus serotypes involved, leading to better detection of outbreaks associated with EV-A71 or any other serotype associated with severe or distinct clinical features.

Technical Appendix Number of sentinel pediatricians and number of children included in surveillance of hand, foot and mouth disease and herpangina, by region, France, April 2014–March 2015.
