# Supplementary material for: Ambulatory Pediatric Surveillance of Hand, Foot and Mouth Disease as Signal of an Outbreak of Coxsackievirus A6 Infections, France, 2014–2015
Source: Emerg Infect Dis. 2016 Nov;22(11):1884–93. doi: 10.3201/eid2211.160590 (PMC5088007; doi:10.3201/eid2211.160590)
Supplement: Technical Appendix — Number of sentinel pediatricians and number of children included in surveillance of hand, foot and mouth disease and herpangina, by region, France, April 2014–March 2015. [file 16-0590-Techapp-s1.pdf]

Article DOI: http://dx.doi.org/10.3201/eid2211.160590

# Ambulatory Pediatric Surveillance of Hand, Foot and Mouth Disease as Signal of an Outbreak of Coxsackievirus A6 Infections, France, 2014–2015

## Technical Appendix

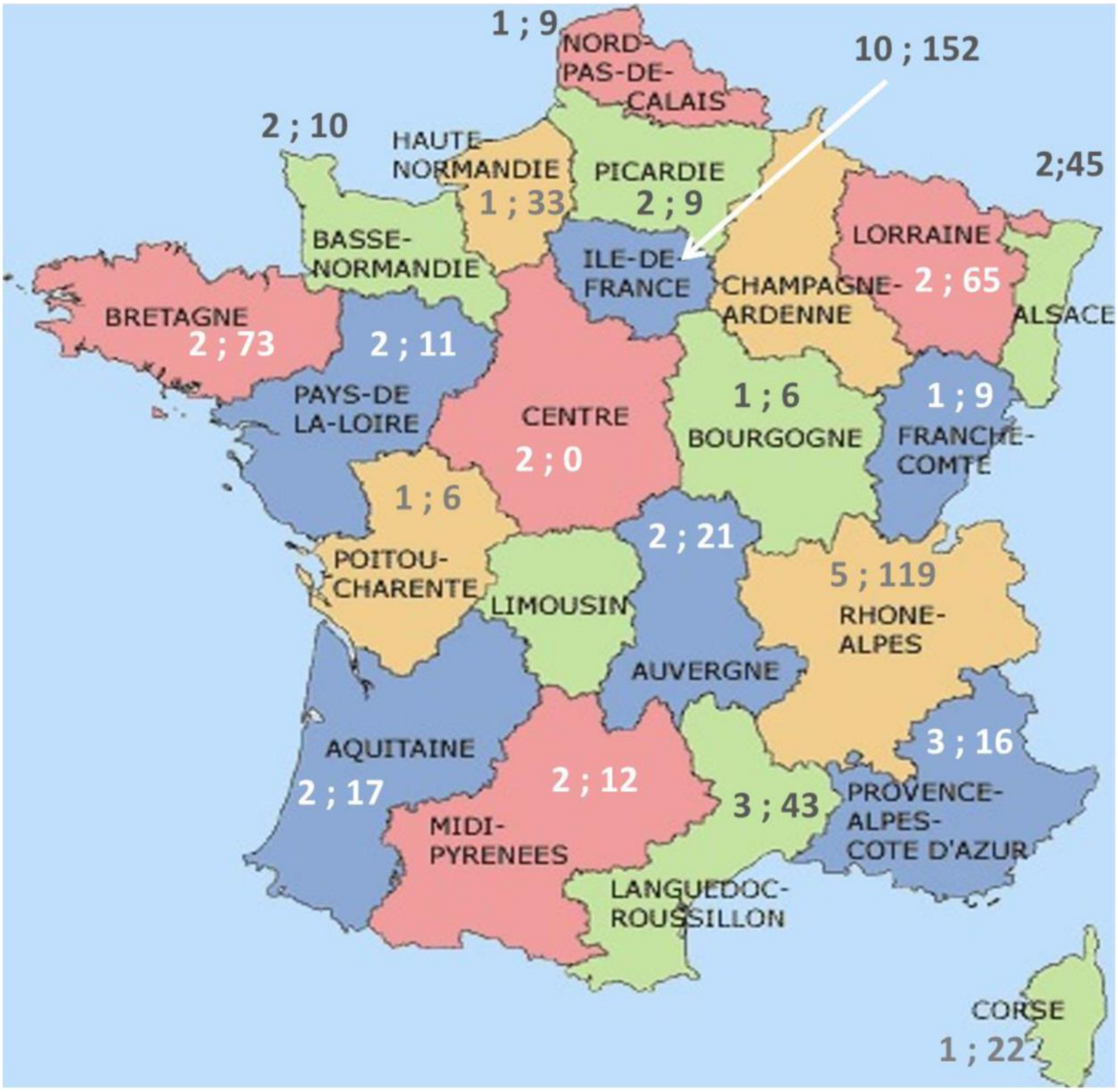


**Technical Appendix Figure.** Number of sentinel pediatricians and number of children included in surveillance of hand, foot and mouth disease and herpangina, by region, France, April 2014–March 2015.
